# Global population-specific variation in miRNA associated with cancer risk and clinical biomarkers

**DOI:** 10.1186/1755-8794-7-53

**Published:** 2014-08-28

**Authors:** Renata A Rawlings-Goss, Michael C Campbell, Sarah A Tishkoff

**Affiliations:** 1 Department of Genetics, University of Pennsylvania, Philadelphia, PA 19104, USA; 2 Department of Biology, University of Pennsylvania, Philadelphia, PA 19104, USA; 3 Department of Biostatistics, Yale University, New Haven, CT 06520, USA

**Keywords:** miRNA, Biomarkers, Population differentiation, Whole-genome sequencing, African genetic diversity, Disease susceptibility, Cancer, Diabetes

## Abstract

**Background:**

MiRNA expression profiling is being actively investigated as a clinical biomarker and diagnostic tool to detect multiple cancer types and stages as well as other complex diseases. Initial investigations, however, have not comprehensively taken into account genetic variability affecting miRNA expression and/or function in populations of different ethnic backgrounds. Therefore, more complete surveys of miRNA genetic variability are needed to assess global patterns of miRNA variation within and between diverse human populations and their effect on clinically relevant miRNA genes.

**Methods:**

Genetic variation in 1524 miRNA genes was examined using whole genome sequencing (60x coverage) in a panel of 69 unrelated individuals from 14 global populations, including European, Asian and African populations.

**Results:**

We identified 33 previously undescribed miRNA variants, and 31 miRNA containing variants that are globally population-differentiated in frequency between African and non-African populations (PD-miRNA). The top 1% of PD-miRNA were significantly enriched for regulation of genes involved in glucose/insulin metabolism and cell division (p < 10^−7^), most significantly the mitosis pathway, which is strongly linked to cancer onset. Overall, we identify 7 PD-miRNAs that are currently implicated as cancer biomarkers or diagnostics: hsa-mir-202, hsa-mir-423, hsa-mir-196a-2, hsa-mir-520h, hsa-mir-647, hsa-mir-943, and hsa-mir-1908. Notably, hsa-mir-202, a potential breast cancer biomarker, was found to show significantly high allele frequency differentiation at SNP rs12355840, which is known to affect miRNA expression levels *in vivo* and subsequently breast cancer mortality.

**Conclusion:**

MiRNA expression profiles represent a promising new category of disease biomarkers. However, population specific genetic variation can affect the prevalence and baseline expression of these miRNAs in diverse populations. Consequently, miRNA genetic and expression level variation among ethnic groups may be contributing in part to health disparities observed in multiple forms of cancer, specifically breast cancer, and will be an essential consideration when assessing the utility of miRNA biomarkers for the clinic.

## Background

MicroRNA (miRNA) expression profiles have been demonstrated to be unique for a wide range of human diseases, including different stages of tumor progression and metastasis [1]. MiRNA expression levels and function can also be affected by global factors, such as genomic variation due to population history, which have been less well studied. MiRNAs function mainly to inhibit protein synthesis through binding between miRNA seed sequences and complementary sequences on target messenger RNA (mRNA) genes. This binding causes degradation and/or translational repression of mRNA genes [2-6]. A single mature miRNA (21–25 base pairs) has the ability to inhibit protein synthesis of over 6,000 mRNA targets [7-13], and miRNAs are predicted to regulate the protein expression of 30–60% of all human protein-coding genes [14,15]. Therefore, changes in miRNA expression in response to disease, the combined effects of circulating miRNA being extremely stable in blood and serum [16] and advances in miRNA detection methods, such as in situ hybridization and RT-PCR, have made miRNAs excellent candidates as diagnostic and prognostic markers in the clinic [17,18]. As a result, miRNAs are currently under clinical investigation as biomarkers for a number of complex diseases, including breast cancer, diabetes (types 1 and 2), asthma, sepsis, lung cancer, prostate cancer, leukemia (ALL and AML), and various pediatric cancers [1,19-27].

**Figure 1:**
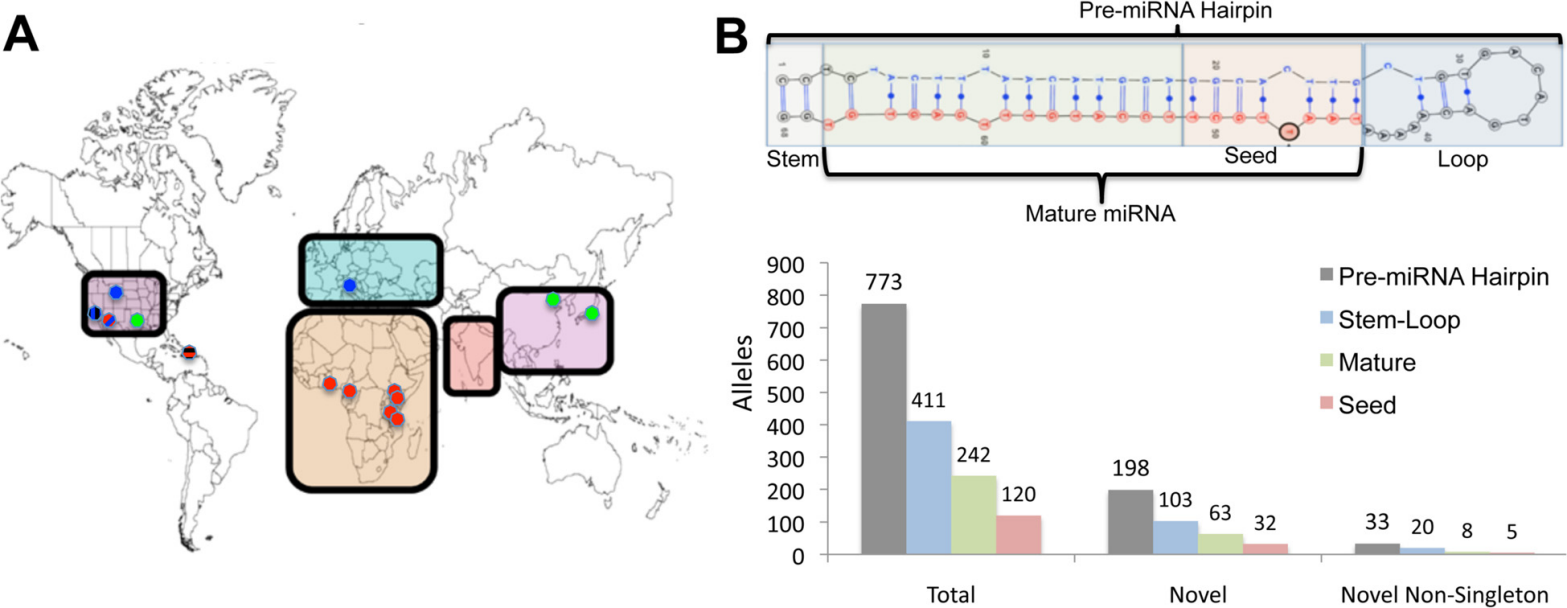
**Variants per miRNA region. (A)** The different colored circles on the world map indicate the geographic location of the 14 sampled populations. Populations from Africa are shown in red (Pygmy, Hadza, Sandawe, Yoruba, Luhya, and Maasai), Asian populations in green (Gujarati Indians, Japanese, and Han Chinese), European populations in blue (Toscani Italians, Utah residents with Northern European ancestry), and recently admixed populations in stripes (African-Americans, Mexican-Americans and Puerto Ricans) (See Methods). Representative regions are enclosed by the thick black lines **(B)** The top panel shows the human miRNA hairpin, hsa-mir-302d, displayed in its modified form with a novel SNP found in its seed sequence circled in black. Seed sequences are defined as the 2^nd^ -8^th^ base of the 5’ end of mature double-stranded miRNA. The mature sequence is highlighted with the guide strand shown in red and the passenger strand shown in blue. The bottom panel depicts the summary of allele types in miRNA genes including novel and non-singleton alleles. Here, mature sequences (green) are defined as all bases within the mature miRNA excluding those within the seed sequence (red). Consistently, stem loops (blue) are defined as all bases excluded from the mature and seed sequences but included in the pre-miRNA hairpin (grey).

Most recent studies identifying potential miRNA biomarkers of disease have been performed in European or Asian populations [28-32] with only a handful of studies performed in populations of African descent [33,34]. Nonetheless, data from these studies have demonstrated that circulating miRNA profiles were considerably different between African-Americans and European-Americans in early stage lung cancer [33] and were expressed differentially between these populations in early stage breast cancer [34]. Given the importance and ubiquitous nature of miRNA-mediated gene expression, it has been proposed that SNPs mapping within miRNA, particularly within the miRNA seed sequences base positions 2–8 of the mature miRNA, may have functional consequences resulting in expression and/or phenotypic variation [4] (Figure 1B). Therefore, genomic variation within miRNA, due to human population history, may be affecting ethnic disparities in complex diseases such as lung and breast cancer through two mechanisms of action: (1) by affecting miRNA expression patterns and (2) by disrupting miRNA/mRNA target recognition through interfering with seed sequence binding.

To date, the genetic coverage of miRNA genes has been low (3× to 5× coverage) in large-scale resequencing projects, such as the 1000 Genomes, which can be problematic for identifying low-frequency variants with high confidence [35]. This is particularly true in African populations which have been shown to possess higher levels of genetic diversity, including low-frequency polymorphisms, compared to other populations worldwide [36-39]. However, diverse African populations are still highly underrepresented in studies of genomic variation [38]. Furthermore, recent studies of population differentiation at miRNA variants have used microarray technology, which captures only a fraction of the total number of miRNA in the genome, resulting in ascertainment bias and also reduced power to discover novel variants [3,40,41].

In the present study, we analyzed 1524 miRNA sequences at high coverage (60×) using whole genome sequence data from 69 individuals representing 14 worldwide populations from Europe, Asia, the Americas and Africa, (Figure 1A) including 3 African hunter-gatherer populations not included in the 1000 Genomes datasets [39]. These samples allow direct comparison of genetic variability in all annotated miRNAs without the ascertainment bias common to SNP array data in geographically and ethnically diverse populations. Based on our data, we identified 33 previously unidentified variants in miRNA genes and 31 significantly population-differentiated (PD) variants based on estimates of F_ST_ between African and non-African populations. We identified 7 PD variants within miRNA that have been experimentally linked to onset, progression, and/or metastasis of cancers with known health disparities between patients of European and African descent. Specifically, we find a T-allele at SNP rs12355840 in hsa-mir-202, that has been shown to increase miRNA expression *in vivo* and to be protective against breast cancer mortality [42], and to be highly PD between African and non-African populations. To our knowledge, a complete survey of genetic variation in all miRNA using high-coverage whole genome data has not previously been performed and has uncovered novel miRNA variants, and determined miRNA biomarker candidates that may differ among diverse population groups.

## Results

### Novel variants identified in miRNA hairpins

In a sample of 69 unrelated individuals, we identified a total of 773 polymorphisms (700 SNPs and 73 insertion-deletions) in pre-miRNA hairpins which passed strict quality control filters (Figure 1B). Of these 773 variants, 411 mutations occurred in pre-miRNA stem/loop regions, 242 in mature miRNA, and 120 in the seed sequences (Figure 1B and Additional file 1: Table S1). Among these polymorphisms, we identified 198 previously undescribed mutations that are currently not present in dbSNP v135. The number of alleles per base was calculated separately for each region of the miRNA (stem-loop, mature, and seed). In our dataset, allele frequencies were slightly higher in the stem-loop region compared to the mature miRNA and seed region (0.013, 0.011 and 0.011, respectively).

To control for somatic mutations, undescribed mutations found in a single individual (ie. singletons) were removed from analyses and 33 novel variants present in multiple individuals (non-singletons) were analyzed (Figure 1B). Among the non-singleton mutations identified in pre-miRNA hairpins, 5 novel mutations were in highly conserved miRNA seed sequences (Figure 1B and Additional file 1: Table S2). The first novel seed variant, a “C/T” SNP at chr3 128081086, was located in the 3’ strand of human miRNA hsa-mir-1280 and was present in one Hadza and one Sandawe, two hunter-gatherer populations from Tanzania. The second, a “C/T” SNP at chr1 62544469, was located in the 3’ strand of hsa-mir-942 and was present in one Hadza and two Sandawe individuals. Two novel seed sequence indels found at low frequency were an “ACA” deletion in miRNA hsa-mir-4483 at chr10 115537763-115537766, found in 2 Yoruban individuals, and a “T”-allele insertion in hsa-mir-3940 at chr19 6416444 in 2 individuals with northern European ancestry from Utah. Lastly, a “CT” deletion located on chromosome 6 in the 3’ strand of hsa-mir-4640 was found in 9 individuals from 7 global populations (namely, 1 Pygmy, 2 Sandawe, 1 Yoruba, 1 Maasai, 2 African-Americans, 1 Mexican-American and 1 Gujarati Indian individual). Using miRNA target prediction software, we determined that in the absence of the “CT” mutation in miRNA hsa-mir-4640, the 3’ strand was predicted to individually target 316 binding sites in 79 genes (Additional file 1: Table S3). With the “CT” deletion, however, the number of predicted targets dropped to 11 binding sites in 3 genes, where gene targets did not overlap between the original and modified miRNA.

### MicroRNA conservation and frequency of population-specific miRNA alleles

**Figure 2:**
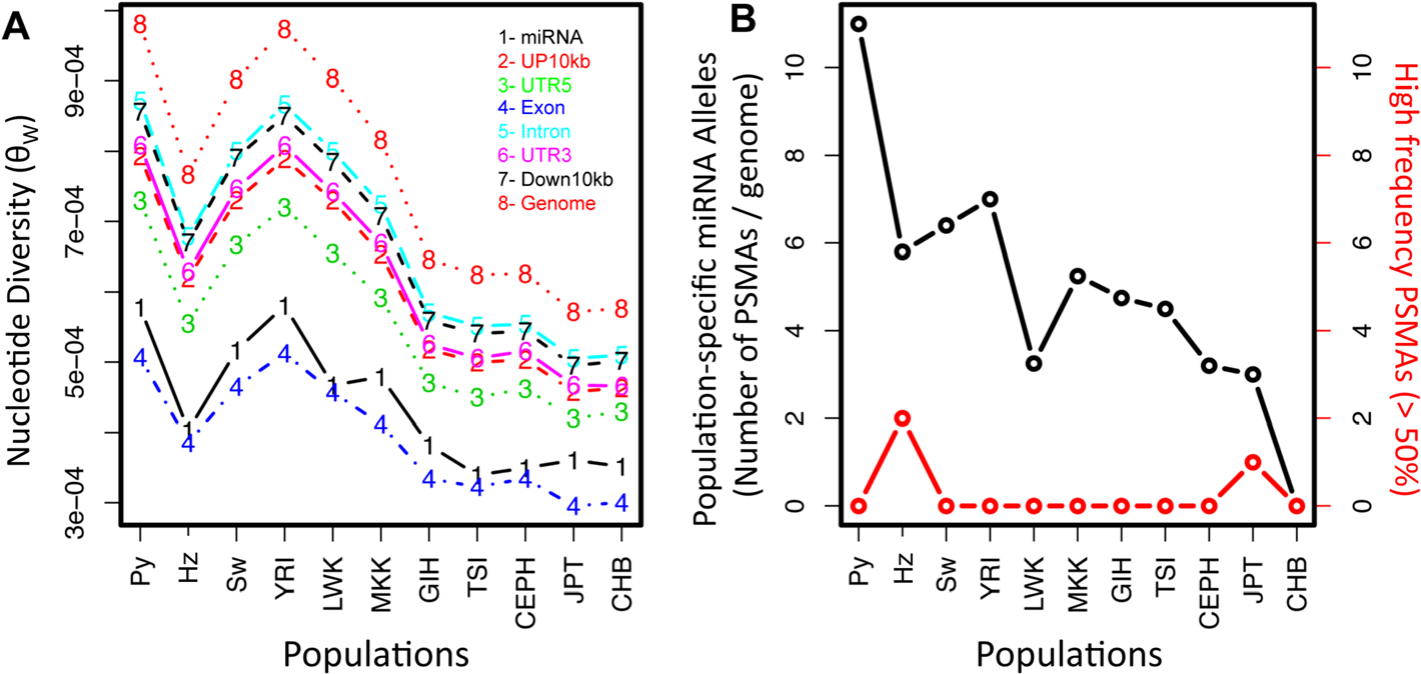
**Population specific SNPs. (A)** Genomic nucleotide diversity, as estimated by Watterson’s (θ), for each population. Within populations, nucleotide diversity was measured for different genomic sequence classes including miRNA genes (black) which were conserved to a level comparable to exons (blue), in all populations. **(B)** The number of population specific miRNA alleles (PSMA) divided by the number of genomes collected for each population (shown in black). The right axis displays the number of high frequency PSMAs, with an allele frequency greater than 50% in a single population (red).

We compared levels of nucleotide diversity in miRNA to other genomic sequence classes (for examples, exons and introns) in each population group using Watterson’s estimator of theta (θ_W_) (Figure 2A). We found in our high-coverage sequence data that miRNAs were among the most conserved sequences in the genome, at the same level as exons (Figure 2A), consistent with results from prior lower-coverage sequencing studies [41]. Overall, however, African populations had the highest level of nucleotide diversity across all sequence classes (θ_W_ = 5.0 ± 0.7 × 10^4^) compared to European and Asian populations (θ_W_ = 3.6 ± 0.2 × 10^4^) (Figure 2A).

In addition, we identified 319 population-specific miRNA alleles (PSMAs), defined as variants present exclusively in 1 of the 14 globally sampled populations. About two-thirds (66.8%) of PSMAs were present in African populations and the proportion of population-specific alleles (ie. population-specific density) was highest in Africans relative to non-Africans, consistent with prior analyses of human genetic variation [41]. In particular, Pygmy hunter-gatherers had the highest population-specific density with 11 PSMAs per genome (Figure 2B). Among the Hadza hunter-gatherers, we found two high frequency PSMAs with allele frequency ≥ 50% in the Hadza only, a novel “A/C” SNP in the stem-loop of hsa-mir-1291 and an “A/G” SNP (rs111566161) in the 3’ mature sequence of miRNA hsa-mir-4711. This “A/G” SNP was found to be exclusively shared by two Hadza and a Southern Kalahari San individual, a population thought to share an ancient genetic ancestry with the Hadza and other African click speaking populations [43,44].

### Population differentiation of human miRNA

**Figure 3:**
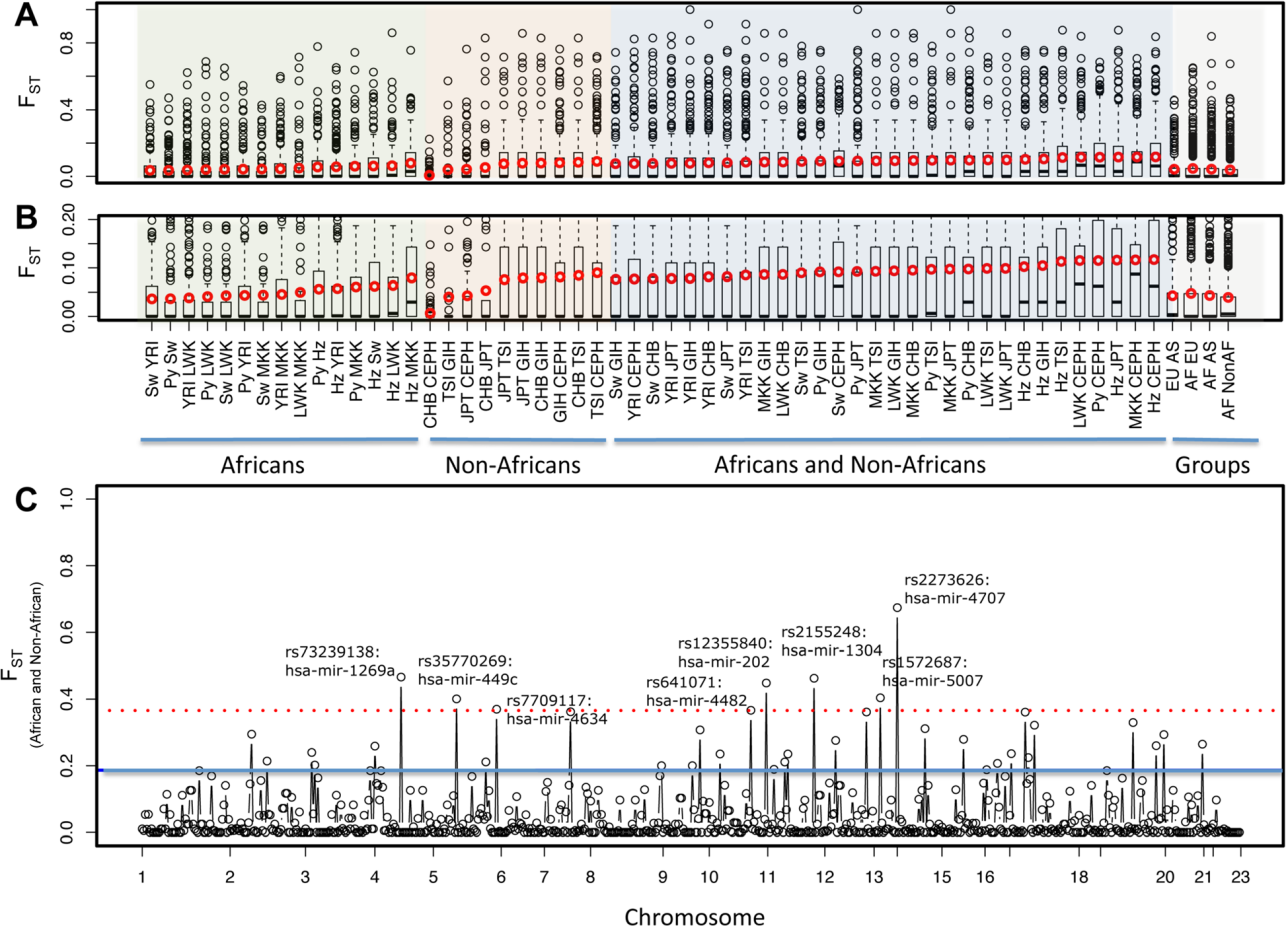
**Pairwise F**_**ST **_**for each miRNA allele. (A)** Boxplots of pairwise F_ST_ between African populations (green), between non-African populations (red), and between African and non-African populations (blue) are grouped hierarchically by major geographic region: Europe-Asia, Africa-Europe, Africa-Asia, and between African and non-African samples (grey). Black circles are outliers above 1.5 times the interquartile range. The red circles and the black solid lines are the mean and median of the distributions, respectively. **(B)** Zoom in of boxplots in A showing the median (black lines) and average F_ST_ of each pairwise group (red circles). **(C)** Pairwise F_ST_ for all miRNA variants between African (n = 32) and non-African (n = 25) samples. Population differentiated miRNAs (PD-miRNAs) contain variants above the 95^th^ percentile (F_ST_ > 0.186) shown as a solid blue line. Highly population differentiated miRNAs (HPD-miRNAs) contain variants above the 99^th^ percentile (F_ST_ > 0.366) shown as a dotted red line and are annotated with miRNA affiliation and rs numbers.

To measure population differentiation, we calculated pairwise F_ST_ at variants in miRNA genes (See Methods). F_ST_ ranges from 0 to 1 with an estimate of 0 indicating no population differentiation and an estimate of 1 indicating complete differentiation. Estimates of F_ST_ at miRNA variants were calculated hierarchically: (a) between individual populations (Figure 3A-B), (b) between major geographic regions (for example, Europe-Africa, Europe-Asia, Africa-Asia) (Additional file 2: Figure S1) and (c) between pooled African and pooled non-African populations (Figure 3C). We then compared the individual estimates of F_ST_ to empirical distributions of miRNA F_ST_. The pairwise F_ST_ values that were outliers (ie. within the top 5% of the empirical distribution or above the 95^th^ percentile) were classified as population-differentiated (PD) and variants with these extreme values were inferred to be enriched for SNPs under recent selection [45]. Our data showed that among major geographic regions, African populations had the highest average F_ST_ values among populations, with 34 PD-miRNA alleles between Africa and Asia, 33 PD-miRNA alleles between Europe and Africa, and 18 PD-miRNA alleles between Europe and Asia (Figure 3B; Additional file 2: Figure S1).

In a comparison of pooled African and pooled non-African populations, we identified 31 PD-miRNAs containing variants with outlier F_ST_ values above the 95^th^ percentile of the distribution of miRNA F_ST_ values (F_ST_ ≥ 0.186 with p < 0.05) (Figure 3C and S2; Additional file 1: Table S4), with 4 PD-miRNA variants in seed sequences (Additional file 2: Figure S3). Furthermore, when we apply a more stringent criteria for population-differentiation among miRNA variants, we found 8 highly population-differentiated miRNAs (HPD-miRNAs) with variants above the 99^th^ percentile representing the top 1% of all pairwise F_ST_ estimates (F_ST_ ≥ 0.366; p < 0.05) (Figure 3C and Additional file 1: Table S4).

### Messenger RNA target and functional enrichment of HPD-miRNAs

**Figure 4:**
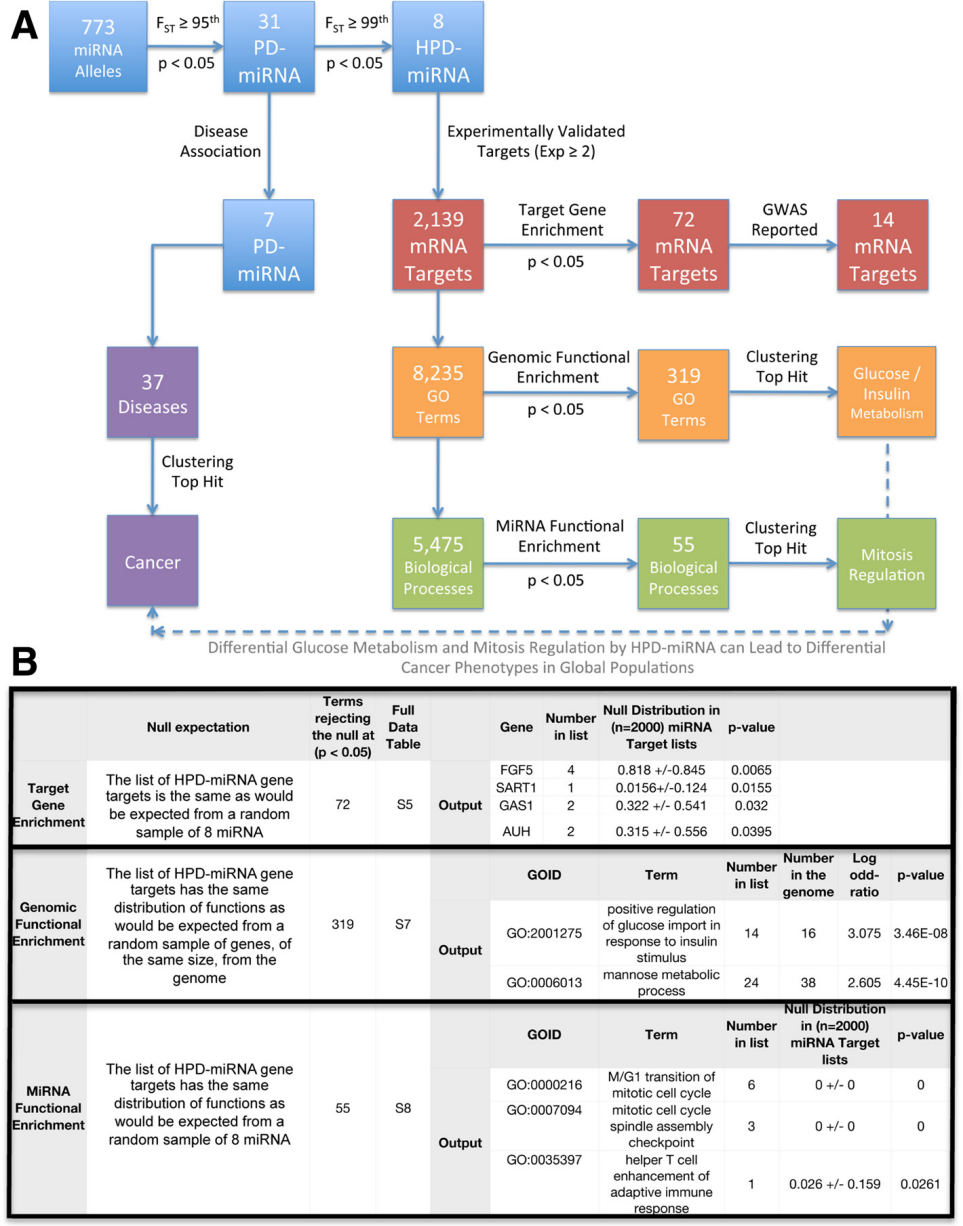
**Analysis flowchart. (A)** Blue boxes represent allele counts, with 773 alleles found in miRNA genes and 31 population-differentiated alleles within miRNA (PD-miRNA). Red boxes represent messenger RNA targets regulated by the 8 highly population-differentiated miRNAs (HPD-miRNAs). These targets were found to be involved in over 8000 gene ontology functions (GO terms) (orange boxes) and over 5,000 biological processes (green boxes). Seven PD-miRNAs were also found to be differentially expressed in 37 diseases, with cancer being the top hit. **(B)** Description of Target enrichment, Genomic Functional Enrichment, and MiRNA functional enrichment that was performed and displayed in **A**. Output of statistical function enrichments were clustered to determine overrepresented functions of HPD-miRNAs. Sugar and insulin metabolism were among the top hits in genomic functional enrichment (p < 0.001), including regulation of the glucose/insulin response pathway. Mitosis was the top hit in miRNA functional enrichment (p <0.001), including regulation of mitosis transition and checkpoint genes by HPD-miRNA.

Experimentally-validated mRNA targets of the 8 HPD-miRNAs were identified by querying 45 publically available deep sequencing and microarray datasets (See Methods). We found that these 8 HPD-miRNAs experimentally down-regulated the expression of 2,139 unique human mRNA target genes in at least two datasets (Figure 4A). Target gene enrichment analysis revealed that 72 of the 2,139 mRNA targets were significantly over-regulated (p < 0.05) by these 8 HPD-miRNAs compared to a null or random set of 8 miRNA (See Methods) (Figure 4A-B and Additional file 1: Table S5). The genes of these over-regulated mRNA targets were involved in immune response, metabolism, developmental processes, cell communication, transport and response to stress (Additional file 2: Figure S4). Additionally, 14 of the 72 enriched gene targets were reported to be candidate loci in genome-wide association studies (GWAS) (Additional file 1: Table S6).

Functional enrichment was performed by two methods for the 2,139 mRNA gene targets of HPD-miRNA. First, genomic functional enrichment was used to determine if particular biological functions regulated by the 8 HPD-miRNAs were overrepresented compared to the null expectation across the genome (Figure 4B) [46]. Based on this analysis, we identified 319 statistically over-represented functions (p < 0.05; n = 319) (Figure 4). The top hits included sugar (mannose, fructose, and glucose) metabolism and the regulation of insulin (p < 10^−7^) (Additional file 1: Table S7). Secondly, we performed a bootstrapping analysis of miRNA functional enrichment to account for the non-random distribution of miRNA targets throughout the genome and identified biological pathways overrepresented by HPD-miRNAs as compared to other non population-differentiated miRNA sets (Figure 4B). In particular, the 2,139 experimentally-validated gene targets of the 8 HPD-miRNAs were found to function in 5,475 annotated biological processes based on the ENSEMBL gene ontology. Each of the 5,475 functions were assigned a probability of being regulated by a random null set of 8 miRNAs (See Methods) and this probability was compared to the observed regulation by the 8 HPD-miRNAs. We found 55 biological processes that were significantly enriched for regulation by our set of 8 HPD-miRNAs (p < 0.05) (Additional file 1: Table S8), and identified 120 HPD-miRNA gene targets involved in enriched biological processes; of particular interest, these targets include *DICER1* and *BRCA1* among other genes (Additional file 1: Table S8).

In addition, clustering analysis of the 55 significant biological processes, based on gene ontology similarity, revealed nine major pathways affected specifically by the 8 HPD-miRNAs: the mitosis pathway, axons and morphogenesis, response to toxins, signaling, acid regulation, transcription, ion sequestration, muscle movement and immune response (Additional file 2: Figure S5). The mitosis pathway contained the highest number of significantly over-represented processes, specifically mitotic transitions (G1 and metaphase/anaphase transition), cell cycle checkpoints, and cytokinesis, representing over 20% of the 55 biological processes. Interestingly, the altered expression of mitotic genes, which can occur through miRNA regulation, is one of the known characteristics of the onset and progression of cancer.

### Disease association analysis reveals links to multiple cancers

**Figure 5:**
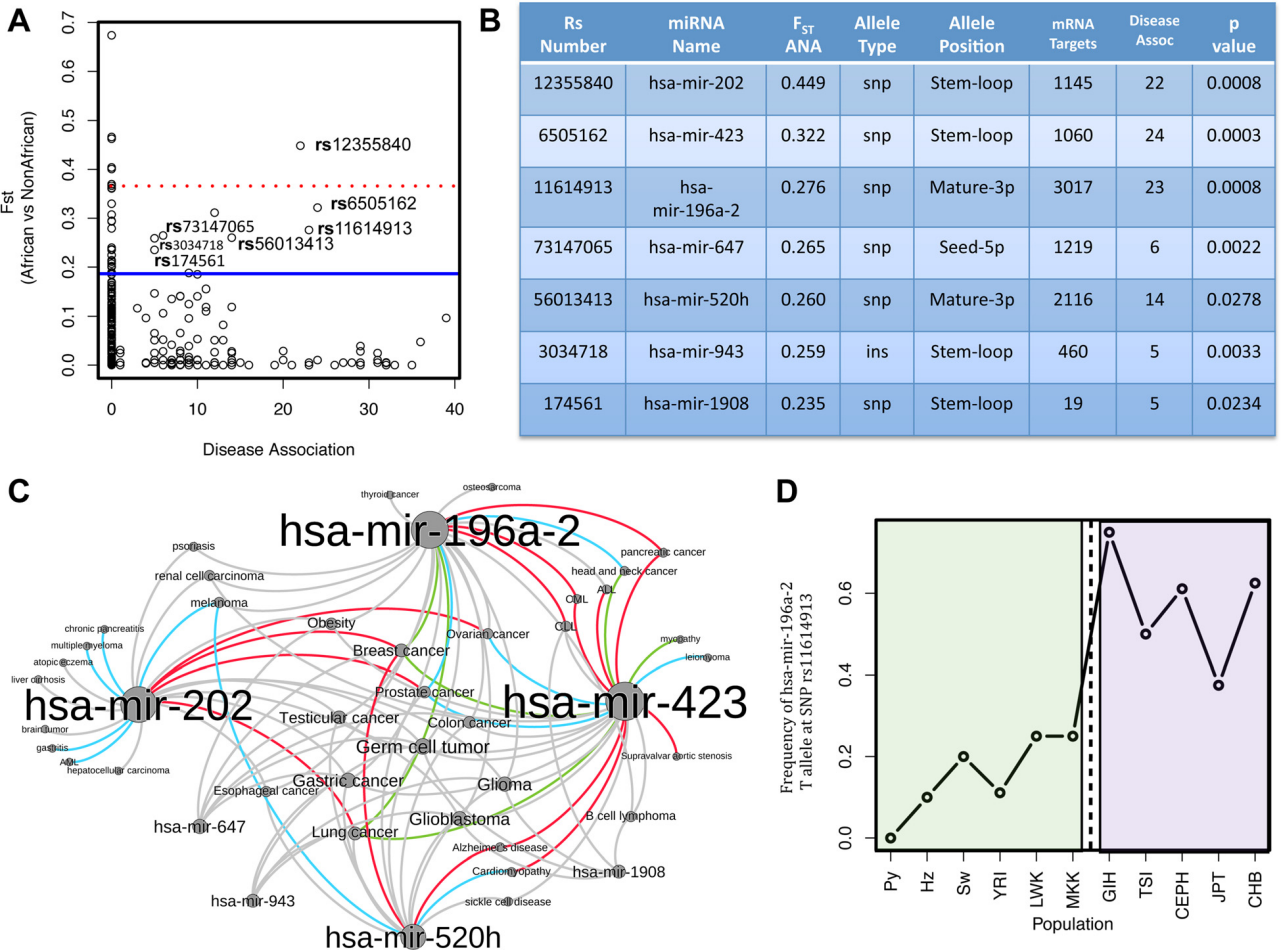
**Disease network analysis. (A)** The number of miRNA disease associations plotted against the pairwise F_ST_ between Africans and non-Africans for each miRNA allele. The 99^th^ percentile (F_ST_ > 0.366) is shown with a dotted red line and the 95^th^ percentile (F_ST_ > 0.186) is shown with a solid blue line. **(B)** Table of F_ST_ values for the 7 PD-miRNAs associated with human diseases and containing alleles with F_ST_ > 95^th^ percentile and p < 0.05. MRNA targets were obtained using 45 public deep sequencing and microarray datasets (See Methods) **(C)** Disease network graph depicting the interactions between the 7 PD-miRNA (shown in black) and human disease, where up-regulation is shown in red, down-regulation (blue), unknown regulation (grey), and miRNA with reports of both up and down regulation in the same disease state shown in green. Labels are scaled by number of disease associations where miRNA or diseases with larger numbers of connecting lines are indicated by larger dot sizes within the network. **(D)** Population allele frequency differentiation of the breast, lung, gastric and esophageal cancer associated T-allele at SNP rs11614913 within miRNA hsa-mir-196a-2. African populations have decreased frequency of the T allele (green) as compared to non-African populations in blue (p < 0.001).

Disease associations were also identified for all 31 PD-miRNAs through the miRNA disease database MiRGator [47] (Figure 5A). Of the 31 PD-miRNAs, 7 miRNAs (hsa-mir-202, hsa-mir-196a-2, hsa-mir-423, hsa-mir-943, hsa-mir-520h, hsa-mir-1908 and hsa-mir-647) were identified in prior studies as being differentially expressed in human diseases representing a significant increase above expectation (1.1 out of 31) for disease-associated miRNAs (95% C.I. = 1.08 – 1.11; p = 2.2 × 10^−16^) (Figure 5B). The 7 PD-miRNAs were associated with 37 diseases, most extensively cancer, with all 7 PD-miRNAs being implicated in cancer risk or as biomarkers for one or multiple cancers, specifically, breast cancer, prostate cancer, testicular cancer, ovarian cancer, lung cancer, renal cell carcinoma, gastric cancer, pancreatic cancer, head and neck cancer, colon cancer, esophageal cancer, brain tumors, thyroid cancer, glioblastoma, glioma, and germ cell tumors (Figure 5C). The hsa-mir-196a-2 T-allele at SNP rs11614913 has been significantly associated with increased risk for esophageal cancer in non-smoking European males [48] but decreased risk for breast, lung and gastric cancers in Chinese populations [48]. In the present study, we observed a significantly lower frequency of the hsa-mir-196a-2 T-allele at SNP rs11614913 in Africans compared to non-African populations (F_ST_ = 0.41; p < 0.001) (Figure 5D).

**Figure 6:**
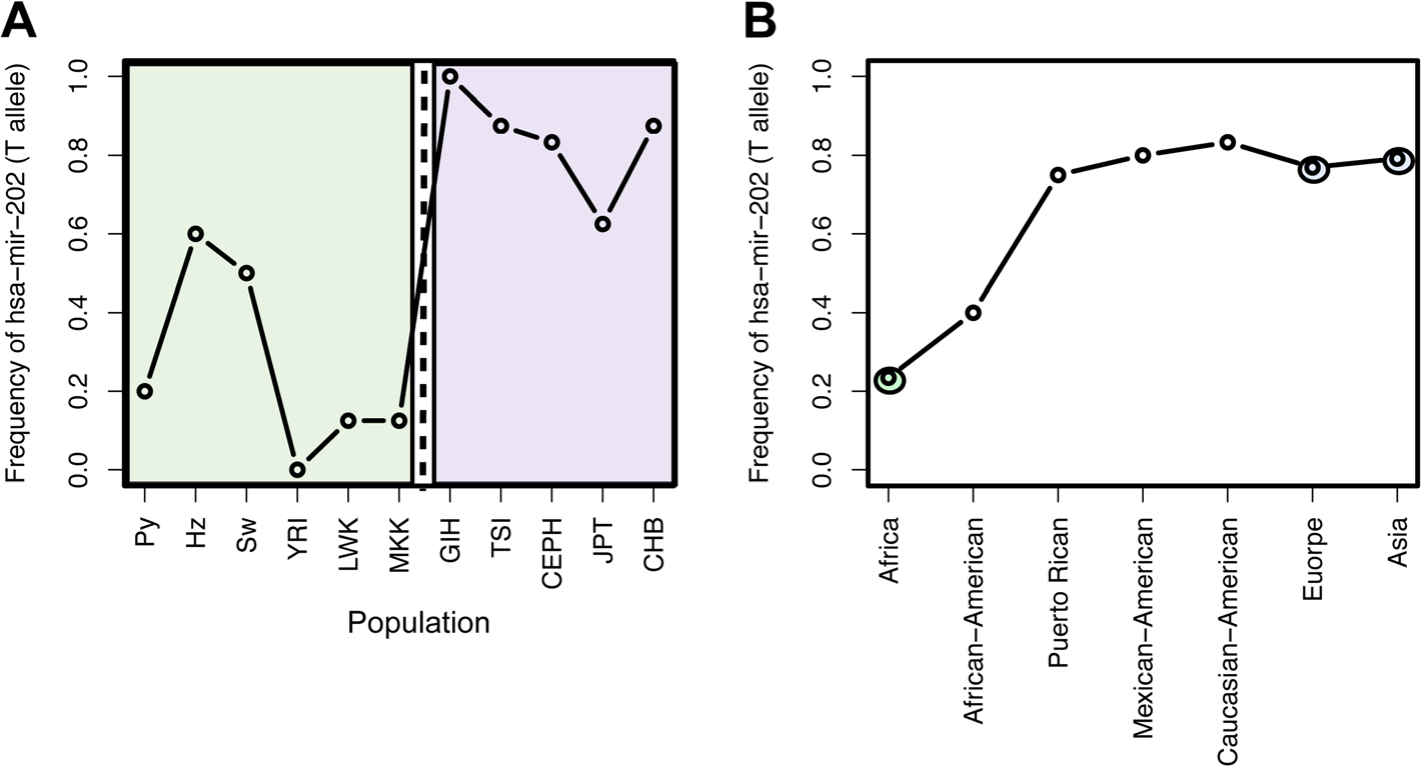
**The role of miRNA hsa-mir-202 SNP in gene expression and breast cancer. (A)** Differences in frequency of the T-allele at mir-202 across populations. African populations are highlighted in green and non-African populations in blue. **(B)** Average frequency of the T-allele at mir-202 in Africa, Europe and Asia (double circles) are compared to a limited sample of US population groups.

Notably, one of our PD-miRNAs, hsa-mir-202, contains a T-allele with known effect on miRNA expression and a protective effect on breast cancer mortality [42]. In our dataset, we found that the frequency of the T-allele at hsa-mir-202 was lower in African populations (26%) compared to non-African populations (65%), on average (Figure 6A and Additional file 1: Table S9). We observed similar frequencies of the T-allele in African and non-African populations in the 1000 Genomes Project data, that sampled only one African population (YRI) (16%) and 2 non-African populations (CEU, CHB + JPT) (83.3% and 92%, respectively) (See Methods). In addition, we also observed variability in the frequency of the T-allele within Africa; specifically, the T-allele occurred at lower frequency in the non-hunter-gatherer Yoruba, Maasai, and Luhya populations ((0.08%) compared to hunter-gather Pygmy, Hadza, and Sandawe populations (43%), on average (Figure 6A and Additional file 1: Table S9). We also found the T-allele at moderate frequency in African-American populations (Figure 6B).

## Discussion

### Population differentiation and functional enrichment of miRNA

Based on analysis of high-coverage whole genome sequence data in 69 individuals from 14 worldwide populations, we identified 33 novel polymorphisms in miRNA genes and 31 variants with high levels of genetic population differentiation between Africans and non-Africans (PD-variants). Five novel variants (2 SNPs and 3 indels) were located in miRNA seed sequences where they could have a large effect on the number, strength, and specificity of each miRNA/mRNA target interaction. Specifically, a novel “CT” deletion found in 7 of the 14 globally diverse populations altered the predicted mRNA targets of hsa-mir-4640 to include 3 additional targets and removed all of its 79 original predicted targets from regulation, indicative of the disruptive potential of seed sequencing indels on miRNA function. In addition, among the 31 PD-variants we found 8 HPD-miRNAs with variants that lie in the top 1% of pairwise estimates of F_ST_, indicating extensive population differentiation at these loci between African and non-African populations consistent with a model of local adaptation (Figure 4) [5,45,49].

From functional enrichment analysis we observe that HPD-miRNAs are significantly enriched for regulation of genes involved in the glucose/insulin metabolism pathway (p < 10^−7^), and cellular division (p < 0.001), notably the regulation of genes involved in mitosis (specifically, mitotic checkpoints, transitions, and cytokinesis) (Figures 4 and 5; Additional file 1: Tables S7 and S8). Aberrant cell division during mitosis or aberrant gene expression levels during chromosome segregation often result in chromosomal instability, which is a key diagnostic feature of most cancers [50]. The disruption of cell division by altering gene expression levels in response to mitotic instability has been strongly correlated with tumor development and progression; both *in vitro* and *in vivo* evidence have demonstrated that in the absence of other cell cycle and DNA repair defects, mitotic disruption can transform cells and predispose them toward cancer [50]. Given that mis-regulation of cellular division is the hallmark of cancer, it is striking that miRNAs with highly population-differentiated alleles are observed to be significantly enriched for regulation of mitotic pathway genes including genes such as *S100A8* and *P2RX3* whose expression profiles are currently used as biomarkers for multiple cancers.

### The Role of miRNA in ethnic disparities in cancer susceptibility

In addition, we identified 7 population-differentiated miRNAs where expression level differences of these PD-miRNAs have been correlated with cancer and other disease phenotypes (Figure 5B-C). Among the identified cancers, higher mortality rates have been reported for breast, ovarian, gastric, prostate and testicular-germ cell cancers in individuals of recent African ancestry compared to individuals of either European or Asian descent (p < 0.001) [51-54]. Of particular interest is hsa-mir-202 which contained one of the most highly population-differentiated variants in our dataset and is one of two miRNAs currently under investigation as a circulating blood-based marker for the detection of non-Hodgkin lymphoma and early stage breast cancer [42,55]. Recent *in vitro* functional data demonstrated that the T-allele was protective against breast cancer mortality by first increasing mature hsa-mir-202 expression levels, leading to subsequent down-regulation of its gene targets, including cancer related genes *CRYBB2, DICER1*, *SART1*, *S100A8, P2RX3,* and *BRCA1*[42]. Diminished expression of mature hsa-mir-202 in individuals harboring at least one non T-allele resulted in a significantly elevated risk of non-Hodgkin lymphoma (OR = 1.83, 95% CI: 1.17–2.85; *P* = 0.008) [42]. Our data showed that African and African American populations had a lower frequency of the T-allele compared to European and Asian populations, suggesting decreased baseline expression levels of mature hsa-mir-202 in African populations. In the context of tissue specific expression, hsa-mir-202 expression varies considerably by tissue type and is more highly expressed in prostate cells compared to breast or ovarian tissues (Additional file 2: Figure S7). Therefore, lower frequencies of the T-allele in African populations have the potential to reduce gene expression levels down below critical thresholds in the breast and ovarian tissues. This reduction may contribute to population differences seen in the onset of breast and ovarian cancer in women of African and European descent (Additional file 2: Figure S7).

Similarly, the other 6 PD-miRNAs identified (hsa-mir-423, hsa-mir-196a-2, hsa-mir-520h, hsa-mir-1908, hsa-mir-647 and hsa-mir-943) have also been implicated in cancer susceptibility in human populations [56]. First, the hsa-mir-423 SNP rs6505162 has been shown to confer reduced risk for breast cancer in women of European decent in GWAS [57]. Second, the hsa-mir-196a-2 SNP rs11614913 CC genotype has been significantly associated with increased risk for breast, lung and gastric cancers in Chinese populations; conversely, the homozygous TT genotype has been significantly associated with esophageal cancer in non-smoking European males [48]. Based on these results, recent studies have called for more detailed analysis of the frequency of this allele in different ethnic groups [58]. We observed a significantly higher frequency of the hsa-mir-196a-2 C-allele at SNP rs11614913 in African compared to non-African populations (F_ST_ = 0.41; p < 0.001) (Figure 5D). Third, hsa-mir-520h expression was determined to be significantly associated with E1A-mediated tumor suppression and cell migration during cancer metastasis and inhibition of hsa-mir-520h significantly decreased the downstream ability of cancer cells to migrate and invade other areas of the body [59]. This pattern has also been observed consistently in different types of cancer including pancreatic, breast and ovarian cancer [59-61]. Also, up-regulation of hsa-mir-520h was shown to increase the effects of the anticancer drug resveratrol in slowing lung cancer tumor mobility [62]. Finally, multiple studies have linked miRNAs hsa-mir-1908, hsa-mir-647 and hsa-mir-943 expression to various cancers known to have ethnic specific disparities [63-65]. Overall, these studies demonstrate that genetic variability within miRNA has the potential to vary miRNA expression and/or mRNA target binding which can be strongly correlated with the onset of multiple cancers, the progression of cancer metastasis and the response to drug therapies. We demonstrate that the frequency of clinically important genomic miRNA variants varies significantly among ethnic populations, particularly between African and non-African groups. Thus, we suggest that population-differentiated variation in miRNA may contribute to ethnic disparities seen in certain forms of cancer.

## Conclusions

Here, we identified several miRNA genetic variants that are highly differentiated among human populations and uncovered a set of HPD-miRNAs that play a role in the suppression, susceptibility, and metastasis of cancer cells. We also found that some of the HPD-miRNA variants are in regions of strong linkage disequilibrium (D' = 1) with markers in a commonly used genotyping array (Illumina 1 M Duo) in African populations that could be included in genome-wide association studies of disease (Additional file 2: Figure S6). Finally, although we focused on population-differentiated miRNAs known to be associated with disease, we also identified an additional 24 PD-miRNAs that represent interesting candidate loci for further study of differential disease risk in ethnically diverse populations. Further investigation is needed in order to understand the patterns of variation at miRNA and their role in phenotypic variation and human adaptation, particularly in African populations which are greatly underrepresented in genomic studies. Additional RNA-sequencing studies, together with eQTL mapping, will be needed in order to assess the effect of PD-variants on gene expression in global populations. Furthermore, future follow-up studies could integrate SNPs within downstream miRNA target sites [66] and upstream miRNA-regulomes (i.e. transcription factors that regulate miRNA genes) [67] with our findings to examine population differentiation or disease association in all phases of the miRNA cycle.

## Methods

### Whole genome sequencing and sample collection

High quality whole genome sequencing (~60× coverage) was obtained for 69 globally diverse individuals from publically-available datasets. Fifteen African hunter-gathers were obtained from Lachance et al. 2012 [39], including 5 Pygmy (Py) (three Baka, one Bakola, and one Bedzan), 5 Hadza (Hz) (plus two technical replicates), and 5 Sandawe (Sw) using the Complete Genomics sequencing platform [68,69].

Additionally, 54 unrelated individuals were obtained directly from Complete Genomics including 9 individuals of Northern European ancestry (combined as CEPH - including 4 CEPH and 5 CEU individuals), 9 individuals of Yoruban ancestry (YRI), 5 individuals of Mexican ancestry (MEX), 5 African-Americans living in Dallas (ASW), and 2 individuals of Puerto Rican ancestry (PUR), and 4 individuals each of Toscani Italians (TSI), Japanese ancestry (JPT), Han Chinese (CHB), Gujarati Indian (GIH), Maasai Kenyan ancestry (MKK), Luhya Kenyan ancestry (LWK) [70]. Variants were defined as autosomal alleles that differ from the human reference genome build (GRCh37/hg19), and novel variants are defined as variants that are absent from dbSNP (db135). Complete Genomic Data is available through their public site: http://www.completegenomics.com/public-data/69-Genomes/.

### Nucleotide diversity and population differentiation

Pre-miRNA locations and mature miRNA sequences were downloaded from the database mirbase (v18) and included 1524 known pre-miRNAs. We filtered known pre-miRNAs into high and low confidence miRNA, based on experimental validation of their function as target suppressors [71]. High confidence miRNAs were classified as those with at least one experimentally-validated mRNA target, using 45 public datasets (See Messenger RNA Target Enrichment and Analysis). Among novel variants, 165 mutations were identified within high confidence miRNA, 83 were found in miRNA stem-loops, 82 in mature miRNA, and 28 in seed sequences. Seed sequence locations were computed in R software and defined as the 2^nd^ – 8^th^ base pair of the mature miRNA [72]. Novel miRNA target prediction was done in DIANA software [73] for hsa-mir-4640 “CT” deletion. Predicted targets were also filtered for those with experimental validation, based on 45 public datasets (See Messenger RNA Target Enrichment and Analysis). Nucleotide diversity for each sequence class was measured using Watterson’s estimator of θ (θ_W_) [74].

Whole genome sequence data were annotated using the UCSC genome browser for sequence class determination. Pairwise population F_ST_ values were calculated using Weir and Cockerham’s weighted equations adjusting for small sample size using the R statistical software [75,76]. MiRNAs were determined to be population differentiated (PD-miRNA) if they contained an allele with a F_ST_ value in the top 95^th^ percentile of the empirical distribution of F_ST_ values (F_ST_ ≥ top 5% with p < 0.05). Highly population differentiated miRNA (HPD-miRNA) were defined as miRNA containing variants with F_ST_ values above the 99^th^ percentile between African and non-Africans (F_ST_ ≥ top 1% with p < 0.05). P-values for F_ST_’s were determined by testing the allele frequency difference at each allele between African and non-African samples using a Welsh two-sided t-test (Additional file 2: Figure S2).

### Messenger RNA target enrichment and analysis

For HPD-miRNA, messenger RNA (mRNA) targets were identified from the consensus of 45 paired miRNA/mRNA experimental datasets, and subject to the following quality control filters: (1) the mRNA targets have to be correlated with miRNA expression with a correlation coefficient of r < −0.5 and (2) that this level of correlation was observed in at least 2 publicly available experimental deep sequencing or microarray datasets. Experimental datasets consisted of: 6 ENCODE (Encyclopedia of DNA elements) cell line RNA sequencing datasets (GM1287- a lymphoblastoid cell line produced from the blood of a female donor with northern and western European ancestry, H1_hESC- a human embryonic stem cell line, Hela_S3- an immortalized cell line from an African-American female patient with cervical cancer, K562- an immortalized cell line from a female patient with chronic myelogenous leukemia (CML), HepG2- a cell line produced from a male patient with liver carcinoma, and NHEK- a epidermal keratinocyte cell line), 13 cancer deep sequencing datasets from the Cancer Genome Atlas (TCGA) (BLCA - Bladder Urothelial Carcinoma, BRCA - Breast invasive carcinoma, COAD - Colon adenocarcinoma, HNSC - Head and Neck squamous cell carcinoma, KIRC - Kidney renal clear cell carcinoma, KIRP - Kidney renal papillary cell carcinoma, LAML - Acute Myeloid Leukemia, LIHC - Liver hepatocellular carcinoma, LUAD - Lung adenocarcinoma, LUSC - Lung squamous cell carcinoma, READ - Rectum adenocarcinoma, STAD - Stomach adenocarcinoma, and UCEC - Uterine Corpus Endometrioid Carcinoma), 2 deep sequencing Gene Expression Omnibus (GEO) datasets (GSE31999 and GSE37765), and 24 microarray datasets from GEO (GSE2564, GSE9234, GSE11255, GSE12250, GSE14224, GSE14473, GSE14794, GSE14834, GSE15387, GSE15745, GSE16558, GSE16654, GSE16759, GSE17306, GSE17491, GSE17498, GSE18155, GSE18693, GSE18899, GSE19350, GSE20692, GSE21032_1, GSE21032_2, GSE21321). Dataset correlation was done through MiRGator v3 software [77].

Two HPD-miRNA (hsa-mir-5007 and hsa-mir-4634) had no targets that passed quality control filters and were excluded from downstream analyses. The number of targets that passed quality control filters for the remaining HPD-miRNA were hsa-mir-202 (1145 targets), hsa-mir-1304 (710 targets), hsa-mir-1269a (488 targets), hsa-mir-4482-1 (144 targets), hsa-mir-449c (69 targets), and hsa-mir-4707 (1 target). Each unique mRNA target was individually analyzed using bootstrapping analysis to test for significant enrichment in HPD-miRNA**.** Specifically, targets were identified for (n = 2000) randomly chosen sets of 8 miRNA. The distribution of random targets was then compared to the 2,139 targets regulated by the 8 HPD-miRNA, and 72 targets were significantly enriched for regulation by HPD-miRNA (p < 0.05) **(**Figure 4 and Additional file 1: Table S5). Enriched targets were then annotated with the PANTHER pathway software (Additional file 2: Figure S4).

MiRNA target prediction was done with and without the novel “CT” deletion observed in hsa-mir-4640 using DIANA prediction software [73]. Canonical hsa-mir-4640 targets were compared among 6 different target prediction algorithms (TargetScan, miRNAorg, Microcosm Targets, PITA, PICTAR and miRDB) using MiRGator v3 software [77].

Experimentally validated gene targets were analyzed for reported genome wide significance in a GWAS study using the NHGRI GWAS catalog (Additional file 1: Table S6) [78]. We determine the extent of linkage disequilibrium (LD) in the regions surrounding the 8 HPD-miRNA SNPs, by using common tagging SNPs from the Illumina 1 M-Duo array in African samples (Additional file 2: Figure S6). The program PHASE v.2.1, which implements a Bayesian statistical method [79], was used to reconstruct multi-site haplotypes from genotype data for 15 SNPs from the Illumina 1 M-Duo SNP array flanking the miRNAs of interest on chromosomes 4, 5, 10,11,13 and 14 in 697 African individuals (Additional file 2: Figure S6). Haploview [80] was then used to calculate pairwise measures of LD among SNP loci, creating a graphical representation of the LD relationships among these loci (Additional file 2: Figure S6).

### Functional enrichment

Genomic functional enrichment was analyzed with GOEAST software to determine statistically overrepresented GO terms within our set of 2,139 mRNA gene targets [46]. GO terms include annotated biological processes, molecular functions, and cellular components. The GOEAST algorithm assumes genes should be evenly distributed across the genome. MiRNA, as a class, may favor targeting a specific range of biological processes. To address this fact we perform miRNA functional enrichment.

MiRNA functional enrichment identifies biological pathways overrepresented by HPD-miRNA as compared to a set of 8 randomly sampled miRNA. We found the 2,139 mRNA gene targets of HPD-miRNA to be involved in 5,475 annotated biological processes using the ENSEMBL Gene Ontology database (Figure 4) [81]. For miRNA functional enrichment, the null distribution was created by, (1) randomly resampling 8 miRNA from the mirbase database, (2) identifying all mRNA targets (meeting the quality control filters described above in Messenger RNA Target Enrichment and Analysis), (3) identifying all biological processes associated with mRNA targets (using the ENSEMBL Gene Ontology database). Steps 1–3 are replicated n = 2000 times. The frequency of obtaining each of the 5,475 biological processes seen in HPD-miRNA is calculated. Finally, the actual frequency of each of the 5,475 biological processes associated with HPD-miRNAs is then compared to the null distribution above to ascertain enrichment of biological processes in our sample. Biological processes overrepresented in HPD-miRNA, having p-values < 0.05, were considered significantly enriched in HPD-miRNA and further clustered by gene ontology similarities using Revigo software [82].

### Disease association

Disease associations were assessed through the MiRGator v2 disease database [47]. Disease associations are defined as differentially expressed miRNA in a published gene expression profile [47]. The significance of disease association results was tested by bootstrapping 31 miRNAs from mirbase and testing for disease association, n = 10,000 times. Network analysis was done using R with disease network visualization performed with Gephi software [83].

## Abbreviations

miRNA: microRNAs; PD-miRNA: Population-differentiated miRNA; HPD-miRNA: Highly population-differentiated miRNA; mRNA: Messenger RNA; SNP: Single nucleotide polymorphism; indels: Insertions and deletions.

## Competing interests

The authors declare that they have no competing interests. Whole genome variant data has been made available through dbSNP, dbGAP and Complete Genomics.

## Authors’ contributions

RR conceived of the study, performed analysis, and wrote the manuscript. MC performed linkage disequilibrium analysis and contributed to manuscript revisions. ST supervised the project and contributed to the writing of the manuscript. All authors read and approved the final manuscript.

## Pre-publication history

The pre-publication history for this paper can be accessed here:

http://www.biomedcentral.com/1755-8794/7/53/prepub

## Supplementary Material

Additional file 2: Figure S1 Pairwise F_ST_ for populations grouped by continent. **Figure S2.** Distribution of p-values for pairwise F_ST_’s measured between African and non-African populations. **Figure S3.** Allele frequencies for the 4 PD-variants in miRNA seed sequences. **Figure S4.** Biological functions of the 72 significant gene targets of HPD-miRNA. **Figure S5.** Linkage disequilibrium plots for HPD-SNPs in African populations based on the Illumina 1M Duo. **Figure S6.** Tissue specific expression of hsa-mir-202. Click here for file

Additional file 1: Table S1 All miRNA variants. **Table S2.** Novel variants within miRNA seed sequences. **Table S3.** Predicted changes in gene targets of hsa-mi-4640-3p with novel "CT" deletion. **Table S4.** All PD-miRNA with F_ST_ above the 95th percentile between African and non-Africans. **Table S5.** Significantly enriched targets of HPD-miRNA. **Table S6.** Significantly enriched genes that were reported in genome-wide association studies. **Table S7.** GOEAST: genetic functional enrichment of the 2,139 mRNA gene targets. **Table S8.** MiRNA functional enrichment of the 2,139 mRNA gene targets. **Table S9.** Allele Frequency for hsa-mir-202 T-allele. T-allele has known effect on miRNA expression and a protective effect on breast cancer mortality [42]. N is the number of chromosomes in each sample group. Click here for file
