# Supplementary material for: Global population-specific variation in miRNA associated with cancer risk and clinical biomarkers
Source: BMC Med Genomics. 2014 Aug 28;7:53. doi: 10.1186/1755-8794-7-53 (PMC4159108; doi:10.1186/1755-8794-7-53)
Supplement: Additional file 2: Figure S1 — Pairwise FST for populations grouped by continent. Figure S2. Distribution of p-values for pairwise FST’s measured between African and non-African populations. Figure S3. Allele frequencies for the 4 PD-variants in miRNA seed sequences. Figure S4. Biological functions of the 72 significant gene targets of HPD-miRNA. Figure S5. Linkage disequilibrium plots for HPD-SNPs in African populations based on the Illumina 1M Duo. Figure S6. Tissue specific expression of hsa-mir-202. [file 1755-8794-7-53-S2.docx]

Supplementary Figures and Tables


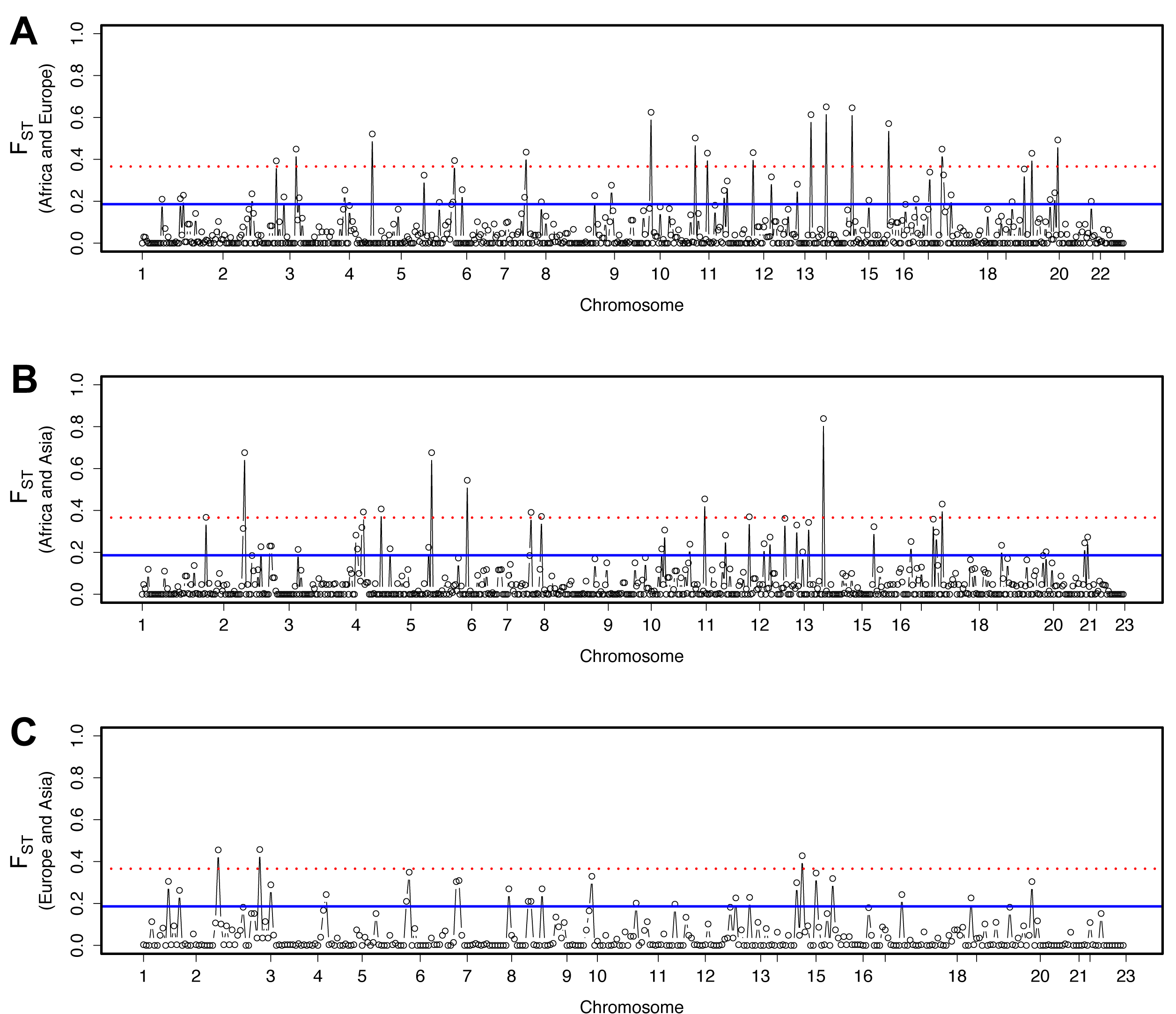


Figure S1: Pairwise F_ST_ for populations grouped by continent (A) between European and African groups (B) between African and Asian groups (C) between European and Asian groups. The 95^th^ percentile and the 99^th^ percentile are represented by blue and dotted red lines, respectively, in each group.


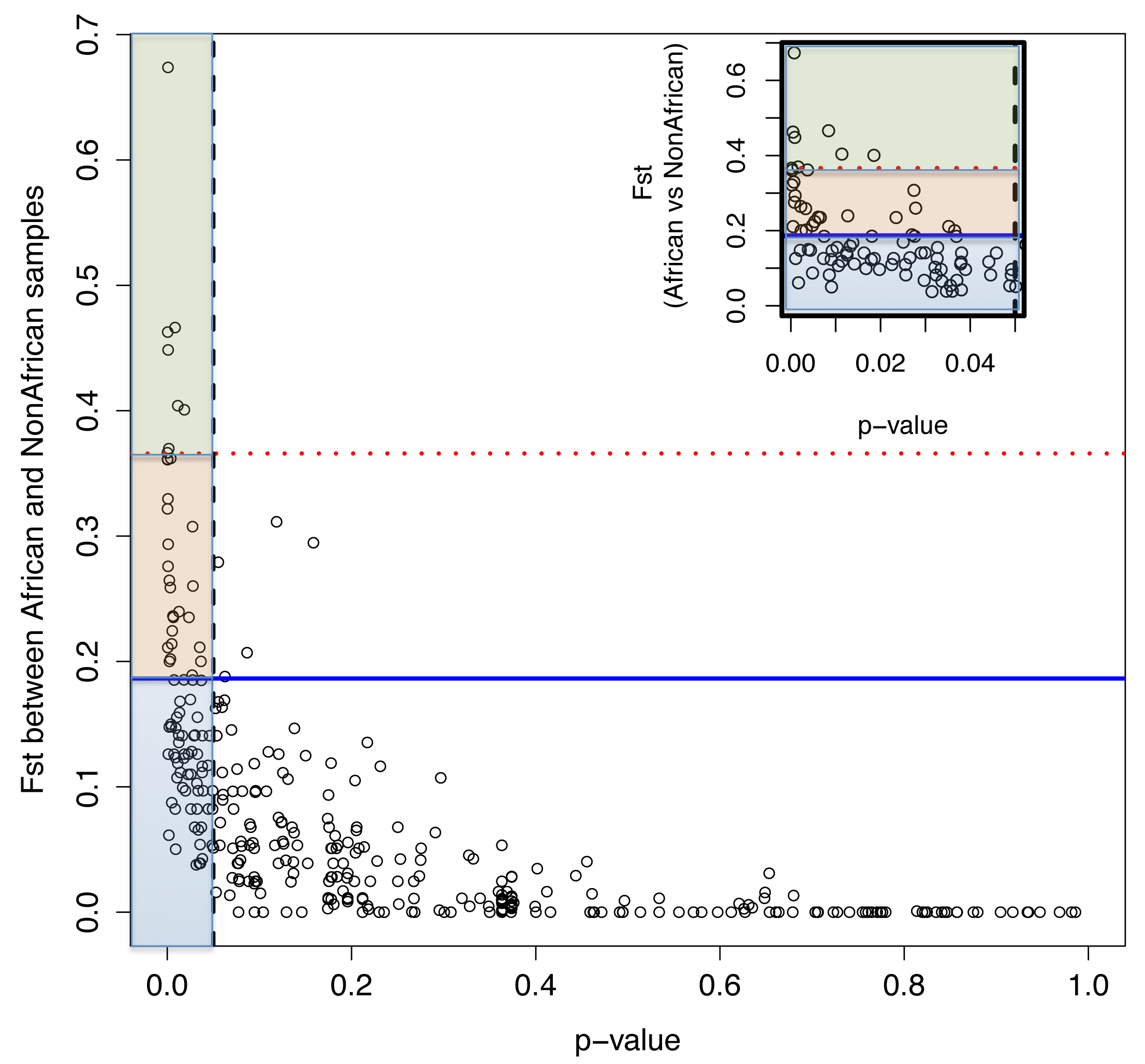


Figure S2: Distribution of p-values for pairwise F_ST_’s measured between African and non-African populations. The 95^th^ percentile and the 99^th^ percentile are represented by blue and dotted red lines, respectively. The black dotted line represents p-values below (p < 0.05). PD-miRNA variants are highlighted in orange and HPD-miRNA variants in green. (Insert) Blow-up of all significant F_ST_’s (p <0.05)


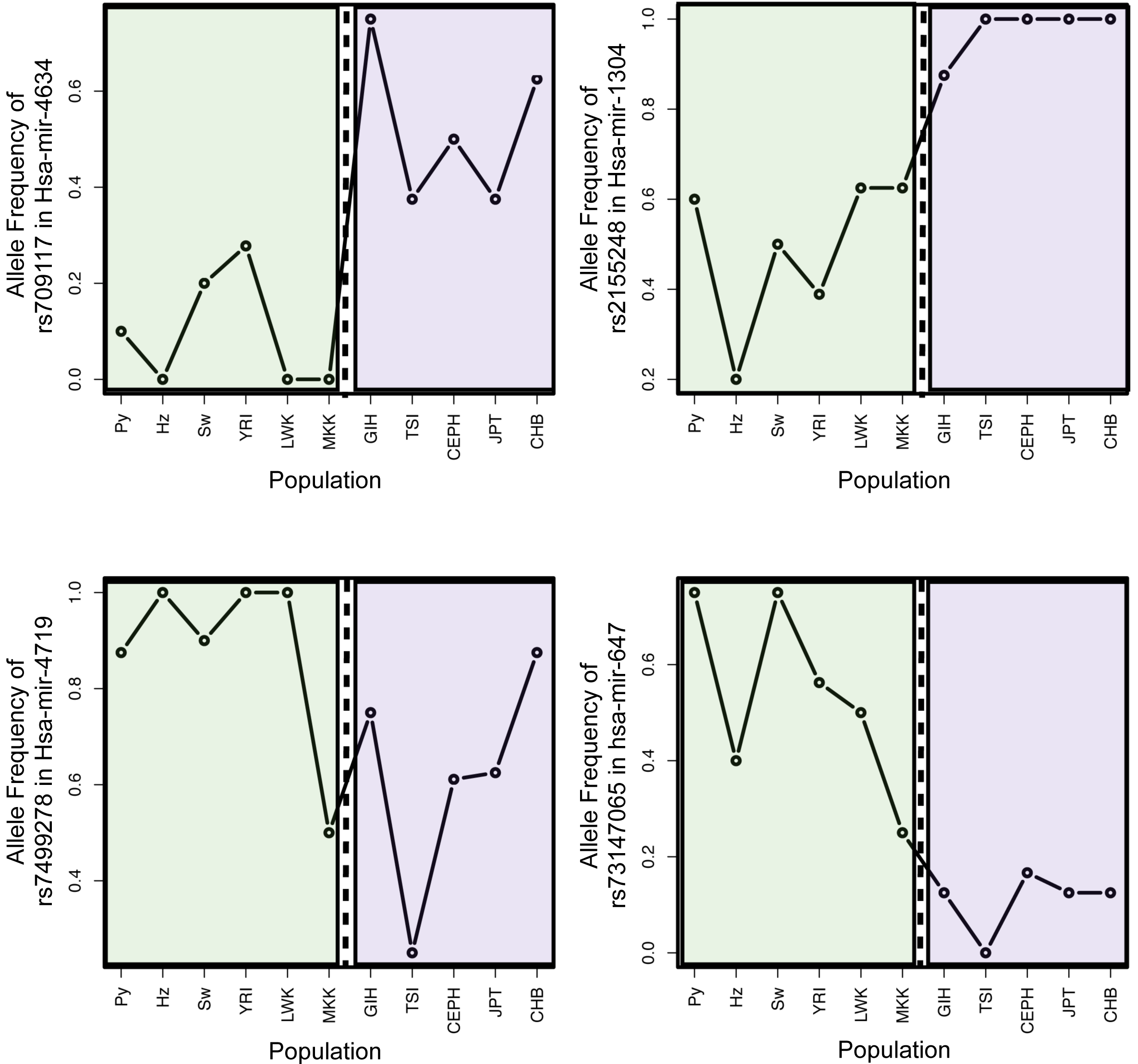


Figure S3: Allele frequencies for the 4 PD-variants in miRNA seed sequences, African populations (green) and non-African populations (blue).


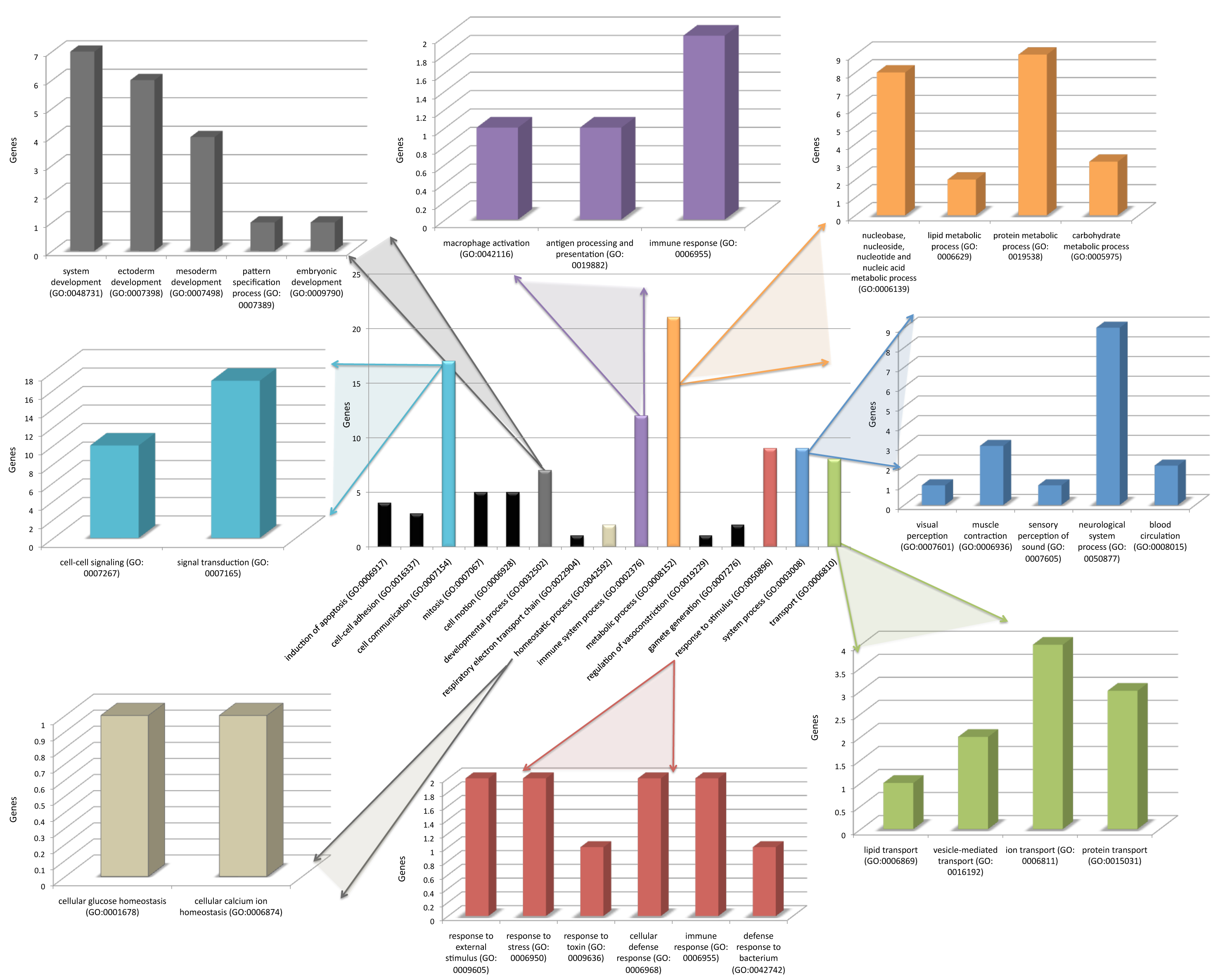


Figure S4: Biological functions of the 72 significant gene targets of the 8 HPD miRNA.


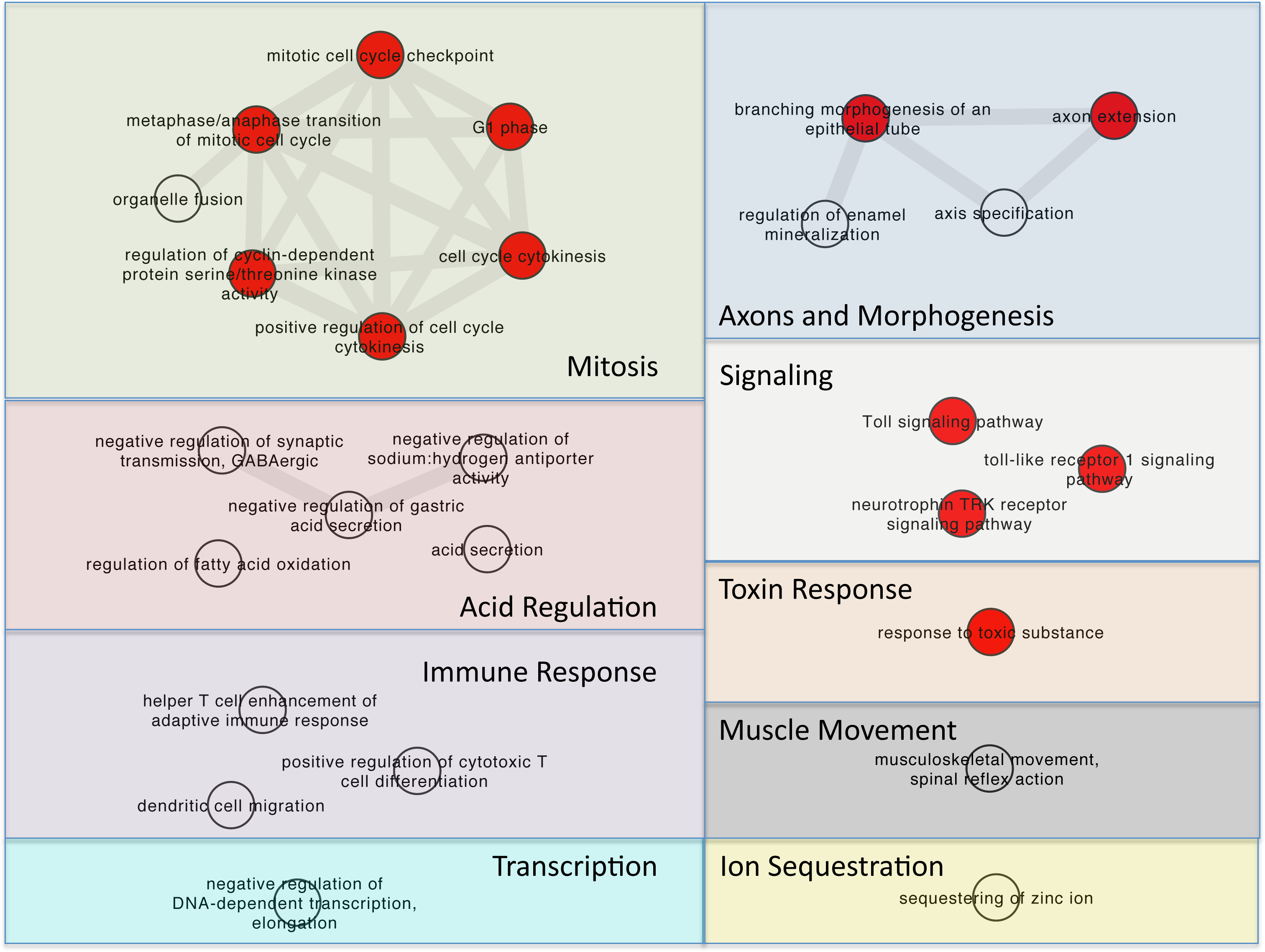


Figure S5: Clustering analysis of enriched biological processes. HPD-miRNA significantly enriched for regulation of 55 biological processes (Supplemental Table S8). Using the REVIGO clustering-like algorithm [[113](#_ENREF_113)], biological processes were clustered based on previously defined measures of semantic similarity in gene ontology [[113](#_ENREF_113)]. Clustering identified 26 semantically unique biological processes that represented the minimum set necessary to describe the entire list. Connected biological processes have similar GO classifications with line thickness representing the strength of the semantic connection. Significance levels of biological process clusters are indicated with bubble color (clusters shown in red have p-values that remain significant after Bonferroni correction).


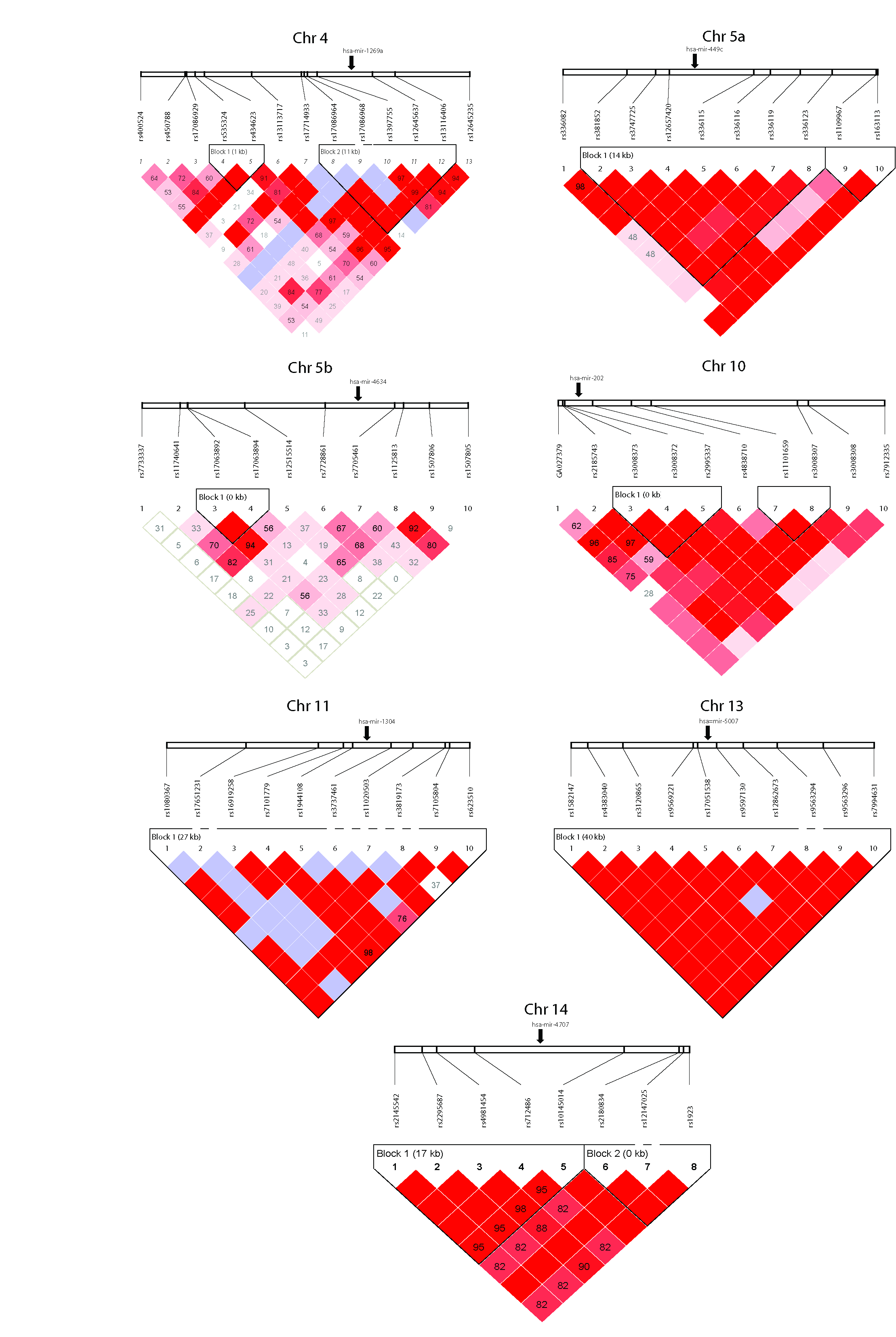


Figure S5. LD plots for HPD-SNPs in African Population from West Central, Central and East Africa. Pairwise plots of LD among genotyped loci from the Illumina 1M Duo SNP array that flank HPD-miRNAs on chromosomes 4,5,10, 11,13 and 14 were constructed using the Haploview program (Barrett et al. 2005). The color of each square signifies the strength of the relationship between SNP alleles. For example, bright red squares indicate statistically significant LD between a given pair of SNP loci (D’=1; LOD > 2), while shades of pink/white squares indicate little evidence for LD. Purple squares indicate high LD but with little statistical support (low LOD). The rs numbers for the 1M Illumina SNPs are given across the top of each plot. The position of the 8 HPD-miRNA SNPs are indicated with a black arrows.


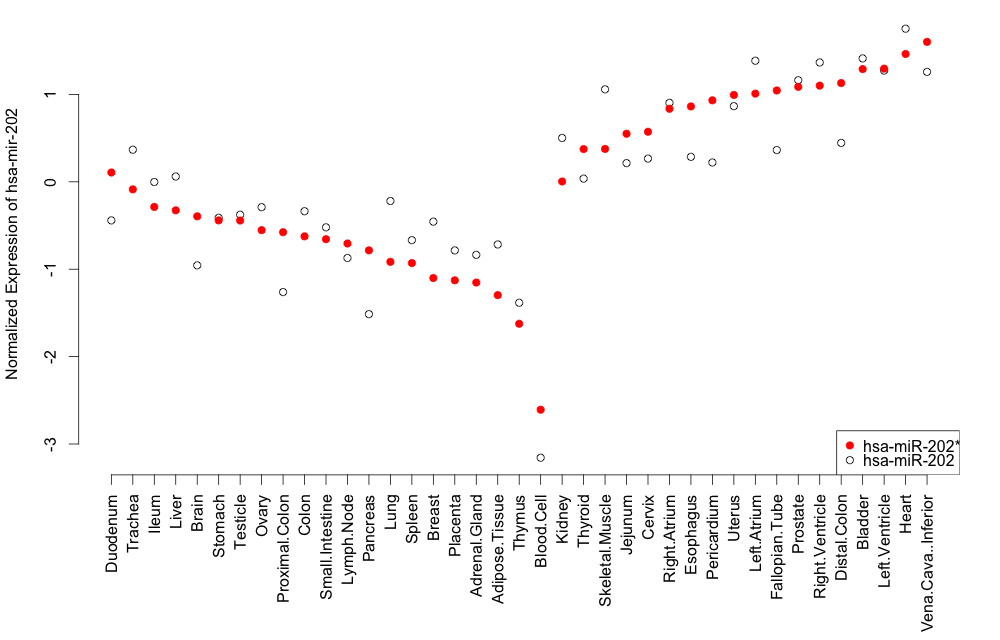


Figure S6: Tissue specific expression of hsa-mir-202. Tissue specific expression was obtained through miRNAbodymap.
